# Trained Immunity in Perivascular Adipose Tissue of Abdominal Aortic Aneurysm—A Novel Concept for a Still Elusive Disease

**DOI:** 10.3389/fcell.2022.886086

**Published:** 2022-05-25

**Authors:** Luca Piacentini, Chiara Vavassori, Gualtiero I. Colombo

**Affiliations:** ^1^ Immunology and Functional Genomics Unit, Centro Cardiologico Monzino IRCCS, Milano, Italy; ^2^ Bioinformatics and Artificial Intelligence Facility, Centro Cardiologico Monzino IRCCS, Milano, Italy; ^3^ Department of Clinical Sciences and Community Health, Cardiovascular Section, University of Milano, Milan, Italy

**Keywords:** abdominal aortic aneurysm, perivascular adipose tissue, immune response, trained immunity, epigenetic markers

## Abstract

Abdominal aortic aneurysm (AAA) is a chronic, life-threatening vascular disease whose only therapeutic option is a surgical repair to prevent vessel rupture. The lack of medical therapy results from an inadequate understanding of the etiopathogenesis of AAA. Many studies in animal and human models indicate a ‘short-circuiting’ of the regulation of the inflammatory-immune response as a major player in the AAA chronic process. In this regard, perivascular adipose tissue (PVAT) has received increasing interest because its dysfunction affects large arteries primarily through immune cell infiltration. Consistently, we have recently produced evidence that innate and adaptive immune cells present in the PVAT of AAAs contribute to sustaining a damaging inflammatory loop. However, it is still unclear how the complex crosstalk between adaptive and innate immunity can be self-sustaining. From our perspective, trained immunity may play a role in this crosstalk. Trained immunity is defined as a form of innate immune memory resulting in enhanced responsiveness to repeated triggers. Specific innate stimuli and epigenetic and metabolic reprogramming events induce and shape trained immunity in myeloid progenitor cells improving host defense, but also contributing to the progression of immune-mediated and chronic inflammatory diseases. Here we present this hypothesis with data from the literature and our observations to support it.

## Introduction

Abdominal aortic aneurysm (AAA) is a chronic and life-threatening vascular disease with a degenerative course leading to rupture of the aorta with fatal consequences in more than 80% of cases. Epidemiologic studies have identified older age, male sex, smoking, family history of AAA, cardiovascular comorbidities (such as ischemic heart disease or peripheral arterial disease), and to lesser extent hypertension and dyslipidemia as the major risk factors associated with the development of AAA (Kent et al., 2010). Patients are mostly asymptomatic and only incidentally are diagnosed for AAA. There are no tests for early diagnosis, nor can the evolution and progression of the disease be predicted. Surgery remains the only treatment option to prevent vessel rupture. The lack of effective noninvasive medical approaches and pharmacological treatments stems substantially from inadequate understanding of the etiopathogenesis of AAA. Despite many efforts over the past decades to understand AAA, it seems quite clear that we are missing some crucial pieces of an intricate puzzle. Therefore, there is an increasing urgency to identify new lines of research that may shed new light on AAA pathogenesis.

## The Pathogenesis of Abdominal Aortic Aneurysm: An Unresolved Tangle

Although historically it was believed that the aneurysm was a final stage of an atherosclerotic process, it is now clear that atherosclerosis and AAA are distinct entities with very specific traits (Tilson, 2016). They share many risk factors, but many aspects are distinctive. Male sex and smoking are, for example., much more prominent risk factors for AAA than atherosclerosis. Low-density lipoprotein cholesterol (LDL-C) is a major determinant of atherosclerosis but has no clear association with AAA. Diabetic patients appear to have a much lower risk of developing AAA than nondiabetic subjects; in contrast, diabetes is one of the major predisposing factors to atherosclerosis and markedly increases the risk of myocardial infarction. Furthermore, therapeutic approaches used for atherosclerotic diseases, such as hypolipidemic, antihypertensive, or antiplatelet drugs, are substantially ineffective in treating AAA (Golledge et al., 2020). The atherosclerotic process can be considered, at least for a subgroup of patients, as a concomitant element in the evolution of AAA but certainly not as a pathophysiological determinant.

### The Immune Response as a Key Element in the Pathogenesis of Abdominal Aortic Aneurysm

A growing number of studies over the past 2 decades support the idea that immune-mediated inflammation plays an important role in AAA development and progression. A chronic immune-mediated response is followed by the destruction of the aortic media through extracellular matrix (ECM)-mediated degradation by the release of proteolytic enzymes (such as metalloproteinases), production of reactive oxygen species (ROS), cytokine action, and vascular smooth muscle cell death, which are typical hallmarks of AAA (Golledge, 2019). The consistent presence of immune-inflammatory cells in tissues of the abdominal aorta has been robustly demonstrated in both animal models and immunohistochemical analyses of human biopsy specimens, suggesting a key role for both innate and adaptive immune cells in AAA evolution. In particular, perivascular adipose tissue (PVAT) has received increasing interest because it has a relevant role in the regulation of vascular physiology and disease, including atherosclerotic and aneurysmal lesions. This multifaceted role of PVAT can be attributed, at least in part, to the fact that PVAT exhibits regional differences and heterogeneity along the vascular tree, e.g., due to the different nature of adipocytes and cellular infiltrates. The heterogeneity of PVAT depots results in a diversity of effects on vascular structure and function, which may influence vessel pathophysiology and contribute to a different manifestation of vascular disease (Gil-Ortega et al., 2015; Queiroz and Sena, 2020). Importantly, PVAT dysfunction is recognized to affect adjacent structures mainly through immune cell infiltration (Guzik et al., 2017). Consistently, we have recently provided evidence that both innate and adaptive immune responses in AAA PVAT contribute to sustaining a damaging inflammatory loop (Piacentini et al., 2019). The underlying assumption is that the inflammatory mechanism may develop from PVAT dysfunction and propagate from the outside to the inside involving the entire thickness of the vessel (Piacentini et al., 2019; Sagan et al., 2019; Gao and Guo, 2022). The presence of cells of adaptive immunity in each layer of the AAA, from the PVAT to the middle of the aorta, has given rise to the hypothesis that the evolution of the AAA may be linked to an antigen-specific response, putatively an autoimmune response, that would feed the chronic inflammatory circuit responsible for disease progression (Chang et al., 2015; Tilson, 2016; Piacentini et al., 2019). Seemingly in contrast to this hypothesis, immunosuppressive treatment approaches tested to date have not produced positive results on AAA outcome, but at times intense immunosuppression has even demonstrated deleterious effects, promoting AAA progression and rupture (Lindeman et al., 2011). One possible explanation is interference with compensatory protection/repair mechanisms, such as protective regulatory T cells, which are responsible for controlling the immune response and have a clear role in limiting AAA growth (Yodoi et al., 2015; Zhou et al., 2015; Suh et al., 2020), or the actions of other cells involved in tissue repair (stem cell function).

All the above implies the involvement of additional as yet unidentified critical factors that do not respond to immunosuppressive chemotherapy and the need to clarify uncertainties about the precise mechanism by which the complex crosstalk between adaptive and innate immunity can be self-sustaining in AAA progression.

### Trained Immunity: a New Concept for Abdominal Aortic Aneurysm

Cells of innate immunity play a key role in AAA because 1) they can perform pathogenically important effector functions on their own, such as responding to stimuli due to tissue damage and secreting soluble factors that degrade the vessel ECM, and 2) they act as mediators of the adaptive response, acting as antigen-presenting cells (exogenous or endogenous) and activating cells of adaptive immunity. Chronic AAA inflammation would thus be a continuous source of stimuli for cells of innate immunity. This would provide the basis for applying a new immunological concept related to innate immunity, so-called ‘trained immunity’, to AAA pathogenesis.

Trained immunity is a term introduced fairly recently in immunology and defines a form of ‘innate immune memory’, which produces an enhanced inflammatory response following secondary stimuli. Stimulation of an innate immune cell (or its progenitors) triggers an enhanced nonspecific immunological reaction as a result of changes in gene transcription caused by modifications in chromatin configuration (Netea et al., 2020). An interplay between epigenetic and metabolic reprogramming events induced by particular innate immune triggers shapes trained immunity in myeloid cells. Among the epigenetic markers that define trained immunity, specific histone modifications have been identified, including histone H3 lysine 4 monomethylation (H3K4me1), trimethylation (H3K4me3), and H3 lysine 27 acetylation (H3K27ac). Furthermore, during the primary challenge, recognition of specific ligands by pattern recognition receptors triggers intracellular cascades that lead to upregulation of metabolic pathways, such as glycolysis, the tricarboxylic acid cycle, and fatty acid metabolism. Several epidemiological and experimental data support the role of trained immunity in the evolution of AAA.

Diet type can positively or negatively influence the development of AAA. Intake of a diet richer in fruits and vegetables is reported to decrease the risk of AAA (Haring et al., 2018) whereas a high-fat diet seems to be associated with an increased risk of AAA rupture, *e.g.,* by accelerating chronic inflammation and altering the physiological functions of PVAT (Hashimoto et al., 2018). Consistently, transcriptomic and epigenomic reprogramming of myeloid progenitor cells that promoted proliferation, skewing of hematopoiesis, and enhanced responses to exogenous and endogenous inflammatory triggers was observed in Ldlr^−/-^ mice fed with the Western diet (Christ et al., 2018).

Long-term changes produced by inflammatory processes that generate alterations in cellular metabolism, epigenome, and transcriptome of bone marrow progenitor cells are known to cause increased myelopoiesis and subsequent expansion of trained innate immune cells (Mitroulis et al., 2018). Consistently, in mouse models of AAA, chronic infusion of angiotensin II caused expansion of hematopoietic progenitors and their differentiation toward proinflammatory CCR2^+^ monocytes (Kim et al., 2016), whereas ablation of the IL-27 receptor protected from aneurysm development by blunting hematopoietic stem cell proliferation and myeloid cell accumulation in the aorta (Peshkova et al., 2019). In the context of AAA, the interaction between activated myeloid cells and a specific T response could be a plausible mechanism to constantly feed the inflammatory loop responsible for the evolution of the dilation process. TLR and NOD signaling pathways could play an important role here both because they have been associated with trained immunity (Owen et al., 2020) and because their involvement in AAA evolution has been extensively documented, including our previous work on the PVAT of AAA (Piacentini et al., 2019; Piacentini et al., 2020).

As mentioned, there is an inverse relationship between diabetes mellitus and AAA. Some studies have suggested that protection is the result of diabetes-induced alterations in the vascular ECM, which make it more resistant to the action of proteolytic enzymes (Dattani et al., 2018). However, important insights have been provided by experimental studies of hypoglycemic drugs in the treatment of AAA. Thiazolidinediones may influence AAA growth by reducing infiltration of innate immune cells (*i.e.*, macrophages) and levels of metalloproteinase and TNFα observed in both the aortic wall and PVAT (Torsney et al., 2013). Of particular interest is the relationship between metformin use and AAA (Raffort et al., 2020). Metformin could exert its anti-inflammatory effect on trained immunity by inhibiting glycolysis through interference with the mTOR/HIF-1α pathway, which is essential for inducing trained immunity (Cheng et al., 2014). The inhibitory effect of metformin on trained immunity provides both an important mechanistic link for a better understanding of AAA pathogenesis and a key clue to its use for the treatment of AAA. Indeed, clinical trials designed to test the efficacy and safety of metformin in preventing the growth of AAA have recently started (Lareyre and Raffort, 2021).

From this complex and varied picture, the possibility arises that the immune response observed in AAAs may have a definite relationship with trained immunity. Therefore, we sought to support this hypothesis by searching for possible associations with epigenetic markers related to trained immunity using the dataset collected on the PVAT of AAAs in our previous study.

## Experimental Evidence

### Study Population and Gene Expression Data

We relied on data collected in a cohort of patients with infrarenal AAA described in (Piacentini et al., 2019). Briefly, the cohort consisted of 30 patients, who underwent elective surgery for AAA at our Centre between June 2010 and December 2014. Patients presented with either a small (<55.0 mm) or large (≥55.0) diameter AAA. The two subgroups did not differ in demographic, clinical characteristics, risk factors, or medications on admission. At the time of surgery, periaortic fat surrounding the aortic neck proximal to the aneurysm (non-dilated-PVAT) and the aneurysm sac (dilated-PVAT) were collected for each patient. We considered the dilated-PVAT and non-dilated PVAT as the pathological and ‘healthy’ (non-diseased) segments of the AAA, respectively. Total RNA from these samples was extracted and used to carry out gene expression analysis with microarrays. Performing a pairwise comparison of dilated-PVAT *vs*. non-dilated-PVAT from each subject, we identified several differentially expressed (DE) transcripts, including 84 and 184 unique genes overexpressed in dilated-PVAT of small and large AAAs, respectively (Piacentini et al., 2019). These genes encompass several factors of the immune-inflammatory response, which appeared relevant for the pathogenesis of AAA (see gene annotation in Supplementary Table S1).

### Bioinformatics Analysis

We used a reverse engineering approach through the *i-cisTarget* software (Imrichova et al., 2015) to analyze the cis-regulatory sequences of genes overexpressed in dilated-PVAT associated with small or large AAAs and predict histone modification signatures. This allowed us to retrieve putative upstream regulators starting from the sets of co-expressed genes and gain insights into functional relationships. The *i-cisTarget* workflow consists of a ‘rank-and-recovery’ procedure. A set of candidate regulatory regions (CRRs) has been established based on publicly available data. For histone modification enrichment, CRRs are scored and ranked according to ChIP-seq features from the ENCODE (Encode Project Consortium et al., 2020) and Roadmap Epigenomics (Roadmap Epigenomics Consortium et al., 2015) projects, including 2450 experimental data tracks. We linked and collected all CRRs located in the proximity of all co-expressed genes in our datasets by setting 10 kb upstream and downstream of the transcription start site (TSS) as the regional mapping boundary and 40% (default) as the minimum overlap fraction of CRRs with peaks. The recovery step extracts the features for which the input foreground regions (the input genes mapped to the CRRs) are most enriched. The enrichment is calculated by the Area Under the recovery Curve (AUC) of these regions. To retain the results of only highly ranked regions, we set the AUC threshold parameter to 1%. The AUCs for all features are then normalized using a Normalized Enrichment Score (NES),

NES=(AUC−μ)/σ

where μ is the mean of all AUC scores across all features and *σ* is the standard deviation of the AUC scores. We set an NES threshold at ≥4.0 to return more stringent results.

The regulatory networks of predicted histone modifications and overexpressed genes in the dilated-PVAT of large and small AAAs related to immune cells were reconstructed using Cytoscape v3.7.1 software (Shannon et al., 2003).

Functional enrichment analysis was performed through Metascape software (Zhou et al., 2019) on groups of genes associated with relevant histone modification marks in small and large AAAs using predefined parameters. Ontology annotation and enrichment were performed using two databases, the Gene Ontology Biological Processes (GO-BP) (Ashburner et al., 2000) and the reference transcription factor (TF)-target interaction database for humans—Transcriptional Regulatory Relationships Unraveled by Sentence-based Text mining (TTRUST) (Han et al., 2018). Significant terms were hierarchically clustered in a tree based on Kappa-statistical similarities (threshold = 0.3) among gene memberships. Representative terms were then visualized as heatmaps.

### Results

**FIGURE 1:**
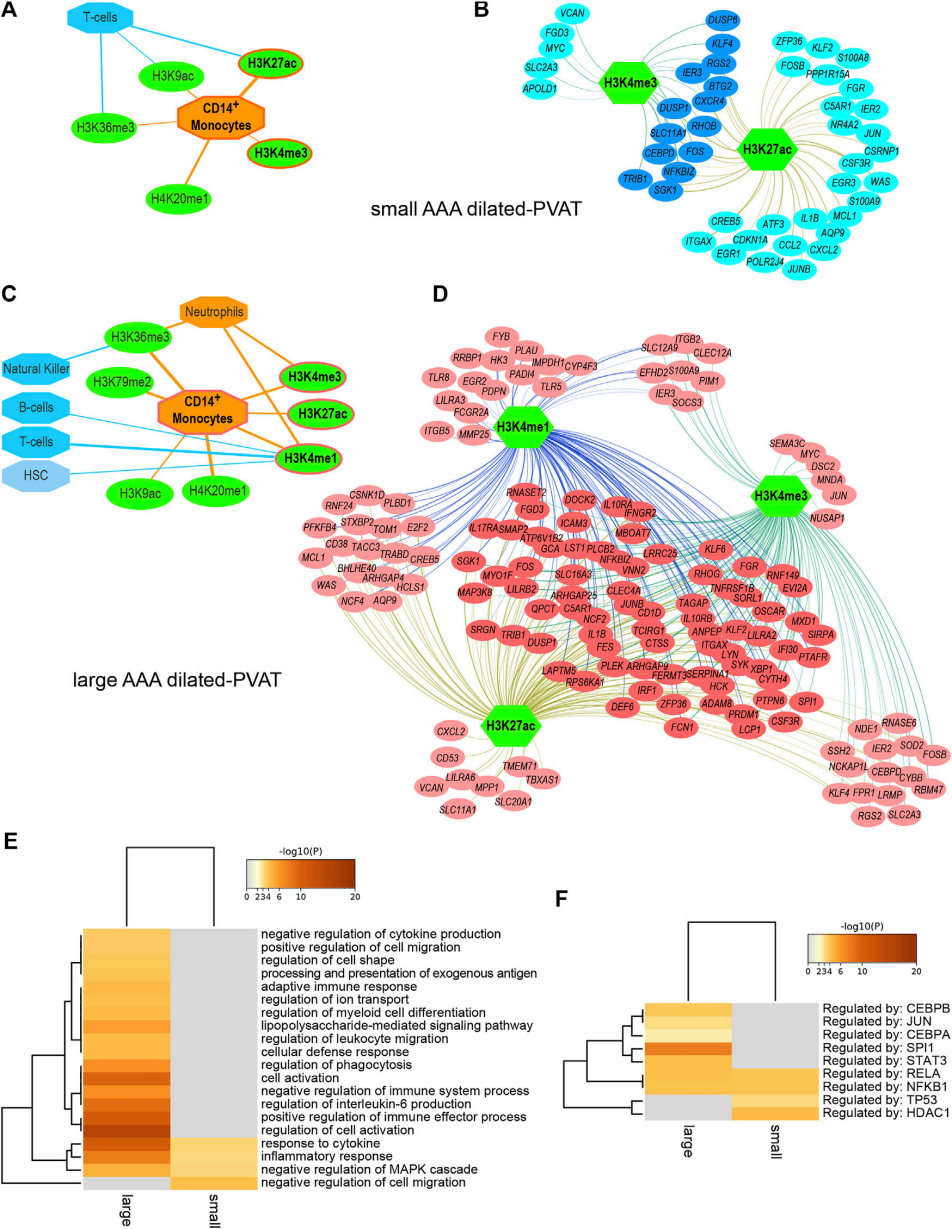
Regulatory networks and functional enrichment analysis of genes overexpressed in AAA dilated-PVAT related to immune cells and functions **(A)** Predicted histone modifications (green ovals) significantly associated with transcriptionally active euchromatin of myeloid or lymphoid immune cells (orange and blue octagons, respectively) in small AAA dilated-PVAT. The histone mark signature recalling immune response after acute stimulation is highlighted with an orange border. In this and panel C, edge thickness is proportional to the normalized enrichment score, which measures the relevance of enrichment **(B)** Regulatory network of histone marks and genes overexpressed in dilated-PVAT linked to acute stimulation in early-stage AAA (small AAA). Green hexagons represent histone marks associated with CD14^+^ monocytes, blue ovals show associated genes. Overexpressed genes associated with this histone mark signature are highlighted in dark blue **(C)** Predicted histone modifications significantly associated with myeloid or lymphoid immune cells in large AAA dilated-PVAT. The histone mark signature recalling trained immunity is outlined in orange **(D)** Regulatory network of histone marks and genes overexpressed in dilated-PVAT linked to trained immunity in advanced AAA (large AAA). Green hexagons are as in panel B, pink ovals show associated genes. Overexpressed genes associated with this histone signature are highlighted in dark pink **(E)** Functional enrichment of Gene Ontology (GO) biological processes and **(F)** transcription factors relative to overexpressed genes associated with acute stimulation or trained immunity. The ochre to brown color gradient indicates the significance level of the GO terms expressed as -log10 of the adjusted *p*-values; gray color indicates non-significant associations. Significant terms were hierarchically clustered based on the Kappa score.

Full results and statistics can be accessed and downloaded from the Zenodo repository (Piacentini and Colombo, 2020). The ‘report’ file for each AAA subtype is an HTML document that can be opened through a browser to explore the analysis parameters and results, including significant features sorted by NES and feature hyperlinks to obtain an overview of significantly high-ranked regions and associated genes. Briefly, reverse engineering analysis allowed us to associate subsets of DE genes with cell type-specific histone modifications. In summary (Table 1), we predicted 18 histone modifications associated with regulatory sequences of subsets of genes overexpressed in the dilated-PVAT of small AAAs and 29 associated with genes overexpressed in large AAAs. Most of these histone modifications were related to immune cells. Specifically, 66% of histone marks in small AAAs were associated with CD14^+^ monocytes, T-helper, and CD8^+^ lymphocytes, while 100% of histone marks in large AAAs were associated with CD14^+^ monocytes, neutrophils, natural killers, hematopoietic stem cells, T-helper, and B-lymphocytes. Most of these histone marks (*i.e.*, H4K20me1, H3K36me3, H3K79me2, H3K4me1, H3K4me3, H3K27ac, and H3K9ac) were related to the transcriptionally active euchromatin of myeloid cells (van der Heijden et al., 2018): indeed, myeloid cell-related histone modifications increased in percentage as AAA diameter increased (small = 39% and large = 72%). This suggested greater transcriptional activity linked to innate immune cells in large AAAs. Finally, we observed that the dilated-PVAT of small and large AAAs was associated with H3K27ac/H3K4me3 and H3K27ac/H3K4me3/H3K4me1 signatures in CD4^+^-monocytes, respectively: the former is suggestive of acute (early) stimulation, the latter is typical of trained immunity (restimulation) (Saeed et al., 2014) (Figures 1A–D).

**TABLE 1:** Predicted histone modifications associated with overexpressed genes in perivascular adipose tissue of small and large abdominal aortic aneurysms.

Feature	Reference	Description	NES
***Small AAA***			
**E124-H3K27ac-broadpeak**	**ENCODE**	**H3K27ac in Monocytes-CD14^+^ RO01746 Primary Cells**	**7.91**
ENCFF001TAS (E124)	ENCODE	H4K20me1 in ChIP-seq on human monocytes CD14^+^	6.47
E127-H3K36me3-broadpeak	ENCODE	H3K36me3 in NHEK-Epidermal Keratinocyte Primary Cells	6.17
E039-H3K27ac-broadpeak	Roadmap	H3K27ac in Primary T helper naive cells from peripheral blood	5.28
E041-H3K36me3-broadpeak	Roadmap	H3K36me3 in Primary T helper cells PMA-I stimulated	5.03
E025-H3K36me3-broadpeak	Roadmap	H3K36me3 in Adipose-derived Mesenchymal Stem Cell cultured cells	4.85
ENCFF001TBR (E126)	ENCODE	H3K9ac in ChIP-seq on human NHDF-Ad	4.79
E124-H3K9ac-broadpeak	ENCODE	H3K9ac in Monocytes-CD14^+^ RO01746 Primary Cells	4.58
E126-H3K9ac-broadpeak	ENCODE	H3K9ac in NHDF-Ad Adult Dermal Fibroblast Primary Cells	4.54
E025-H3K9ac-broadpeak	Roadmap	H3K9ac in Adipose-derived Mesenchymal Stem Cell cultured cells	4.52
E119-H3K36me3-broadpeak	ENCODE	H3K36me3 in HMEC Mammary Epithelial Primary Cells	4.33
ENCFF001TAQ (E124)	ENCODE	H3K36me3 in ChIP-seq on human monocytes CD14^+^	4.31
E124-H3K9ac	ENCODE	H3K9ac in Monocytes-CD14^+^ RO01746 Primary Cells	4.27
E034-H3K27ac-broadpeak	Roadmap	H3K27ac in Primary T cells from peripheral blood	4.21
E047-H3K9ac-broadpeak	Roadmap	H3K9ac in Primary T CD8^+^ naive cells from peripheral blood	4.16
E042-H3K36me3-broadpeak	Roadmap	H3K36me3 in Primary T helper 17 cells PMA-I stimulated	4.05
E124-H4K20me1-broadpeak	ENCODE	H4K20me1 in Monocytes-CD14^+^ RO01746 Primary Cells	4.01
**ENCFF001TAL (E124)**	**ENCODE**	**H3K4me3 in ChIP-seq on human monocytes CD14** ^**+**^	**4.00**
***Large AAA***			
ENCFF001TAS (E124)	ENCODE	H4K20me1 in ChIP-seq on human monocytes CD14^+^	7.86
E029-H3K36me3-broadpeak	Roadmap	H3K36me3 in Primary monocytes from peripheral blood	7.52
E124-H4K20me1-broadpeak	ENCODE	H4K20me1 in Monocytes-CD14^+^ RO01746 Primary Cells	7.31
E037-H3K4me1-broadpeak	Roadmap	H3K4me1 in Primary T helper memory cells from peripheral blood 2	6.69
E124-H3K79me2-broadpeak	ENCODE	H3K79me2 in Monocytes-CD14^+^ RO01746 Primary Cells	6.66
E030-H3K4me1	Roadmap	H3K4me1 in Primary neutrophils from peripheral blood	6.53
**E124-H3K4me1-broadpeak**	**ENCODE**	**H3K4me1 in Monocytes-CD14** ^**+**^ **RO01746 Primary Cells**	**6.42**
E030-H3K4me1-broadpeak	Roadmap	H3K4me1 in Primary neutrophils from peripheral blood	6.37
**E124-H3K4me3**	**ENCODE**	**H3K4me3 in Monocytes-CD14** ^**+**^ **RO01746 Primary Cells**	**6.34**
E030-H3K36me3-broadpeak	Roadmap	H3K36me3 in Primary neutrophils from peripheral blood	6.09
E030-H3K4me3	Roadmap	H3K4me3 in Primary neutrophils from peripheral blood	6.03
ENCFF001TAQ (E124)	ENCODE	H3K36me3 in ChIP-seq on human monocytes CD14^+^	5.97
E040-H3K4me1-broadpeak	Roadmap	H3K4me1 in Primary T helper memory cells from peripheral blood 1	5.46
**E124-H3K27ac-broadpeak**	**ENCODE**	**H3K27ac in Monocytes-CD14** ^**+**^ **RO01746 Primary Cells**	**5.25**
E124-H3K79me2	ENCODE	H3K79me2 in Monocytes-CD14^+^ RO01746 Primary Cells	5.24
E046-H3K36me3-broadpeak	Roadmap	H3K36me3 in primary Natural Killer cells from peripheral blood	5.12
E029-H3K27ac	Roadmap	H3K27ac in Primary monocytes from peripheral blood	5.12
E124-H3K9ac	ENCODE	H3K9ac in Monocytes-CD14^+^ RO01746 Primary Cells	4.83
E043-H3K4me1-broadpeak	Roadmap	H3K4me1 in Primary T helper cells from peripheral blood	4.78
E034-H3K4me1-broadpeak	Roadmap	H3K4me1 in Primary T cells from peripheral blood	4.76
ENCFF001TAR (E124)	ENCODE	H3K79me2 in ChIP-seq on human monocytes CD14^+^	4.64
E124-H3K27ac	ENCODE	H3K27ac in Monocytes-CD14^+^ RO01746 Primary Cells	4.62
E030-H3K36me3	Roadmap	H3K36me3 in Primary neutrophils from peripheral blood	4.57
E031-H3K4me1-broadpeak	Roadmap	H3K4me1 in Primary B cells from cord blood	4.44
E050-H3K4me1	Roadmap	H3K4me1 in Primary hematopoietic stem cells G-CSF-mobilized female	4.42
E029-H3K4me1-broadpeak	Roadmap	H3K4me1 in Primary monocytes from peripheral blood	4.38
E031-H3K4me1	Roadmap	H3K4me1 in Primary B cells from cord blood	4.35
E124-H3K4me1	ENCODE	H3K4me1 in Monocytes-CD14^+^ RO01746 Primary Cells	4.26
E029-H3K36me3	Roadmap	H3K36me3 in Primary monocytes from peripheral blood	4.24

AAA, abdominal aortic aneurysm; ENCODE, Encyclopedia of DNA Elements; Roadmap, NIH Roadmap Epigenomics Consortium.

Histone mark signatures for acute (early) or trained immunity stimulation in small and large AAA, respectively, are highlighted in bold.

To search for possible biological processes associated with gene signatures of acute (early) or trained immunity, we performed a functional enrichment analysis and identified statistically enriched GO-BP and TF terms. To reduce redundancy, terms were grouped based on similarity and an overview term was used for clustering (Figure 1E and Supplementary Table S2). Interestingly, the “inflammatory response” and “response to cytokines” processes were enriched in both the small and large dilated-PVAT, but with higher enrichment scores for the large AAA, suggesting a higher inflammatory state for this subtype. Furthermore, large AAA showed significant associations with several immune-related processes, including “positive regulation of immune effector process”, “regulation of IL-6”, “cellular activation”, and “regulation of myeloid cell differentiation”, suggesting an increase in the immune-related inflammatory process occurring in later stages of the disease. Consistently, we found enriched TFs that are known to be involved in the innate and inflammatory immune response (Figure 1F) such as RELA and NFKB1 in both small and large AAAs and a specific association of large AAAs with CEBPB and SPI1 (PU.1) TFs, which have recently been shown to be associated with trained immunity (de Laval et al., 2020).

## Discussion

AAA is still a disease of unknown etiology that requires new ideas to improve our ability to understand its pathogenetic mechanisms so that effective therapeutic approaches can be developed. In this perspective article, we aimed to highlight possible links between the development and progression of AAA and innate immunity. The existence of innate immunological memory has only recently emerged as a new and rapidly growing immunological concept in several areas, including cardiovascular disease, although its association with AAA has not yet been appreciated. We pointed out that trained immunity is an immunological mechanism capable of generating an excess response when dysregulated, which could contribute to chronic inflammatory disease processes in AAA.

A large number of studies have unequivocally demonstrated that innate and adaptive immunity participate together in the evolution of AAA (Yuan et al., 2020). Furthermore, PVAT has been shown to play a key role in generating the immune-inflammatory response in AAA. Our previous studies corroborate this hypothesis, suggesting that both innate and adaptive immunity in the PVAT of AAAs cooperate to promote an “immunological short circuit” likely responsible for disease progression. Accordingly, through bioinformatics analysis, we previously reconstructed a regulatory network by retrieving transcription factors and upstream regulators (e.g., kinases) of DE genes that characterize dilated AAA PVAT (Piacentini et al., 2020). In the present work, we focused on epigenetic regulators of gene expression, looking for possible histone modifications of pathogenic AAA PVAT genes. Thus, we predicted the histone modification signature associated with trained immunity in the PVAT of large AAAs, which supports the idea that innate immunity may concur with adaptive immunity in consistently feeding the chronic process during later stages of AAA.

Whether elements of innate immunity may be more central than adaptive immunity or *vice versa* is, however, a mere speculative disquisition because both arms of the immune system exert their function through close crosstalk. Consistent with this view, a local environment created by trained innate immune cells during secondary stimulation may indeed influence T-cell responses in AAA, for example by altering the differentiation, polarization, and function of T-cell subtypes, suggesting an important link between trained immunity and an antigen-specific immune response (Murphy et al., 2021).

In this scenario, therefore, it may make sense to test whether trained immunity is a sensitive target that could interrupt this “short circuit” and influence disease evolution. The precise definition of the molecular mechanisms underlying trained immunity in AAA could lead to the identification of possible modulators capable of inhibiting inflammation and vessel wall degeneration, opening new therapeutic solutions for AAA (Arts et al., 2018). To this end, a more thorough investigation using single-cell resolution techniques would be extremely informative in providing insights into the main cell types and their states responsible for the pathophysiology of AAA.

## Data Availability

The original gene expression dataset on which this study is based can be found in the NCBI’s GEO repository at https://www.ncbi.nlm.nih.gov/geo/query/acc.cgi?acc=GSE119717. Overall results and statistics have been made publicly available on the Zenodo Open Science data repository at https://zenodo.org/record/4139226#.X5rzaVDSJPY.

## Supplementary Material

Supplementary Table S1 Annotation of genes significantly overexpressed in dilated-PVAT vs non-dilated-PVAT of large or small abdominal aortic aneurysms or both.

Supplementary Table S2 Functional enrichment results using Gene Ontology (GO) biological process (BP) terms on genes associated with acute and trained immunity histone modification signatures.
